# A case study in applying artificial intelligence-based named entity recognition to develop an automated ophthalmic disease registry

**DOI:** 10.1007/s00417-023-06190-2

**Published:** 2023-08-03

**Authors:** Carmelo Z Macri, Sheng Chieh Teoh, Stephen Bacchi, Ian Tan, Robert Casson, Michelle T Sun, Dinesh Selva, WengOnn Chan

**Affiliations:** 1 Discipline of Ophthalmology and Visual Sciences, The University of Adelaide, Adelaide, South Australia Australia; 2 Department of Ophthalmology, The Royal Adelaide Hospital, Adelaide, South Australia Australia

**Keywords:** Named entity recognition, Electronic health records, Artificial intelligence, Registry, Case study, Application, Tool

## Abstract

**Purpose:**

Advances in artificial intelligence (AI)-based named entity extraction (NER) have improved the ability to extract diagnostic entities from unstructured, narrative, free-text data in electronic health records. However, there is a lack of ready-to-use tools and workflows to encourage the use among clinicians who often lack experience and training in AI. We sought to demonstrate a case study for developing an automated registry of ophthalmic diseases accompanied by a ready-to-use low-code tool for clinicians.

**Methods:**

We extracted deidentified electronic clinical records from a single centre’s adult outpatient ophthalmology clinic from November 2019 to May 2022. We used a low-code annotation software tool (Prodigy) to annotate diagnoses and train a bespoke spaCy NER model to extract diagnoses and create an ophthalmic disease registry.

**Results:**

A total of 123,194 diagnostic entities were extracted from 33,455 clinical records. After decapitalisation and removal of non-alphanumeric characters, there were 5070 distinct extracted diagnostic entities. The NER model achieved a precision of 0.8157, recall of 0.8099, and *F* score of 0.8128.

**Conclusion:**

We presented a case study using low-code artificial intelligence-based NLP tools to produce an automated ophthalmic disease registry. The workflow created a NER model with a moderate overall ability to extract diagnoses from free-text electronic clinical records. We have produced a ready-to-use tool for clinicians to implement this low-code workflow in their institutions and encourage the uptake of artificial intelligence methods for case finding in electronic health records.


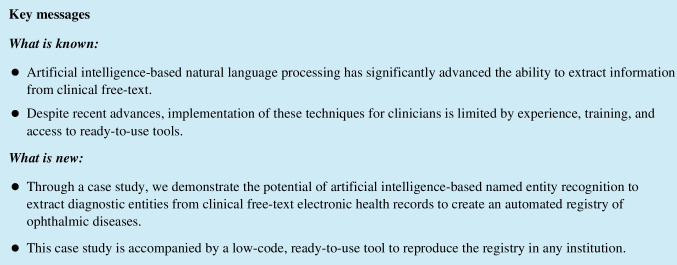


## Introduction

Artificial intelligence-based natural language processing (NLP) techniques have significantly improved the ability to extract information from free text [1]. This technology has important implications for improving the recording of diagnoses in electronic health records. Supplementing manually coded diagnoses with those found in text improves patient cohort identification in studies involving the secondary use of electronic health records [2]. However, applying new and advanced artificial intelligence methods for diagnostic named entity recognition (NER) requires expert knowledge of these techniques and the skills to implement them. These skills are unfamiliar to most clinicians and are a significant barrier to implementing NLP in clinical and research workflows.

Artificial intelligence-based methods have advantages over the previous dictionary and rule-based techniques for clinical named entity recognition. Dictionary-based approaches such as the clinical Text Analysis and Knowledge Extraction System (cTAKES) are early examples of NLP that provided good performance for NER with clinical text. The cTAKES algorithm implemented terminology-agnostic dictionary look-up within a noun-phrase look-up window [3]. However, dictionary-based approaches are limited by the uniqueness of biomedical vocabulary, including abbreviations [4–6], misspellings [7], variable representations of similar concepts [8], ambiguity [9], and variable representations of numbers in text [10]. Rule-based approaches can often achieve high performance [11, 12], but are practically limited by needing to be tailored to specific entities and texts, which restricts generalizability, and are resource intensive requiring extensive expert knowledge and time to develop. Powerful feature-engineered supervised machine learning methods such as conditional random fields (CRF) and support vector machine algorithms further improved the performance of NER beyond dictionary and rule-based approaches, demonstrating the potential application of machine learning to natural language processing and increasing their use [13]. Deep learning methods, including neural networks, have shown additional increases in performance [14, 15]. In particular, recurrent neural networks have shown examples of superior performance to CRF for clinical text [1]. More recent advancements in transfer learning and transformer-based models have improved performance even further [15]. Artificial intelligence offers more generalisable approaches to disease identification without extensive clinician input.

Despite a general awareness of the uses of artificial intelligence, clinicians’ lack of artificial intelligence training and experience may present a barrier to implementing such technology [16–18]. Education of clinicians regarding artificial intelligence and assistance with implementation is an emerging priority [19], given that clinicians will be a critical factor in adopting AI in healthcare. Developing artificial intelligence-based tools and workflows that are easy to use, production-ready, and low-code may assist in facilitating the introduction of artificial intelligence techniques into healthcare and research. There are few ready-to-use tools to apply to clinical text for diagnostic registry production using clinical NER [15]. Thus, we sought to develop and demonstrate the application of low-code artificial intelligence-based NLP tools applied to electronic clinical records to build an automated registry of ophthalmic diseases.

## Methods

We performed this study at the Royal Adelaide Hospital, Adelaide, Australia, with the approval of the institutional Human Research Ethics Committee, adhering to the tenants of the Declaration of Helsinki. We extracted deidentified free-text ophthalmology clinic records from the EHR system for all adult outpatient ophthalmology clinics between November 2019 and May 2022. All notes were free text and written in English.

**Fig. 1:**
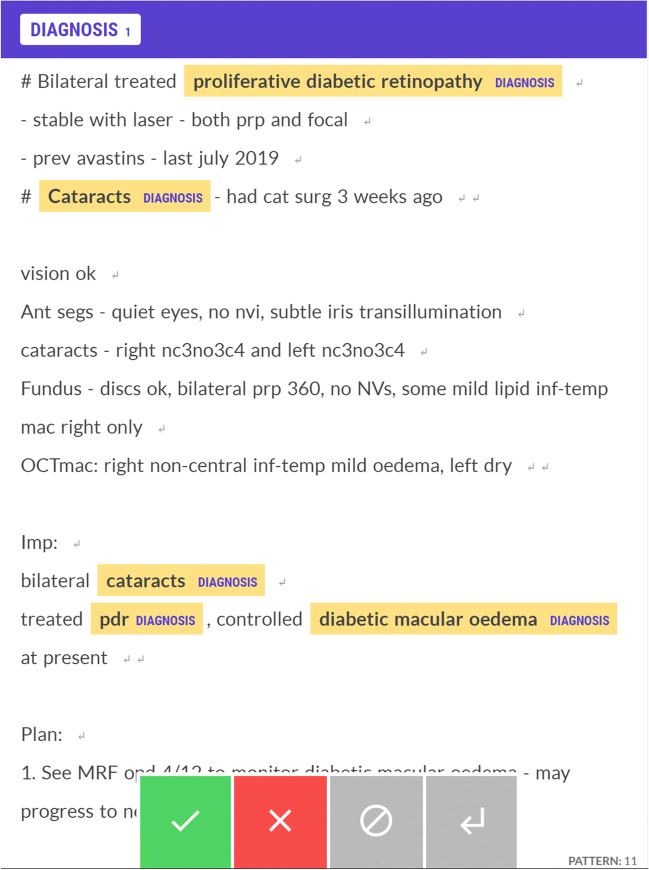
The graphical user interface for annotation dataset creation with example annotations of diagnostic entities

**Fig. 2:**
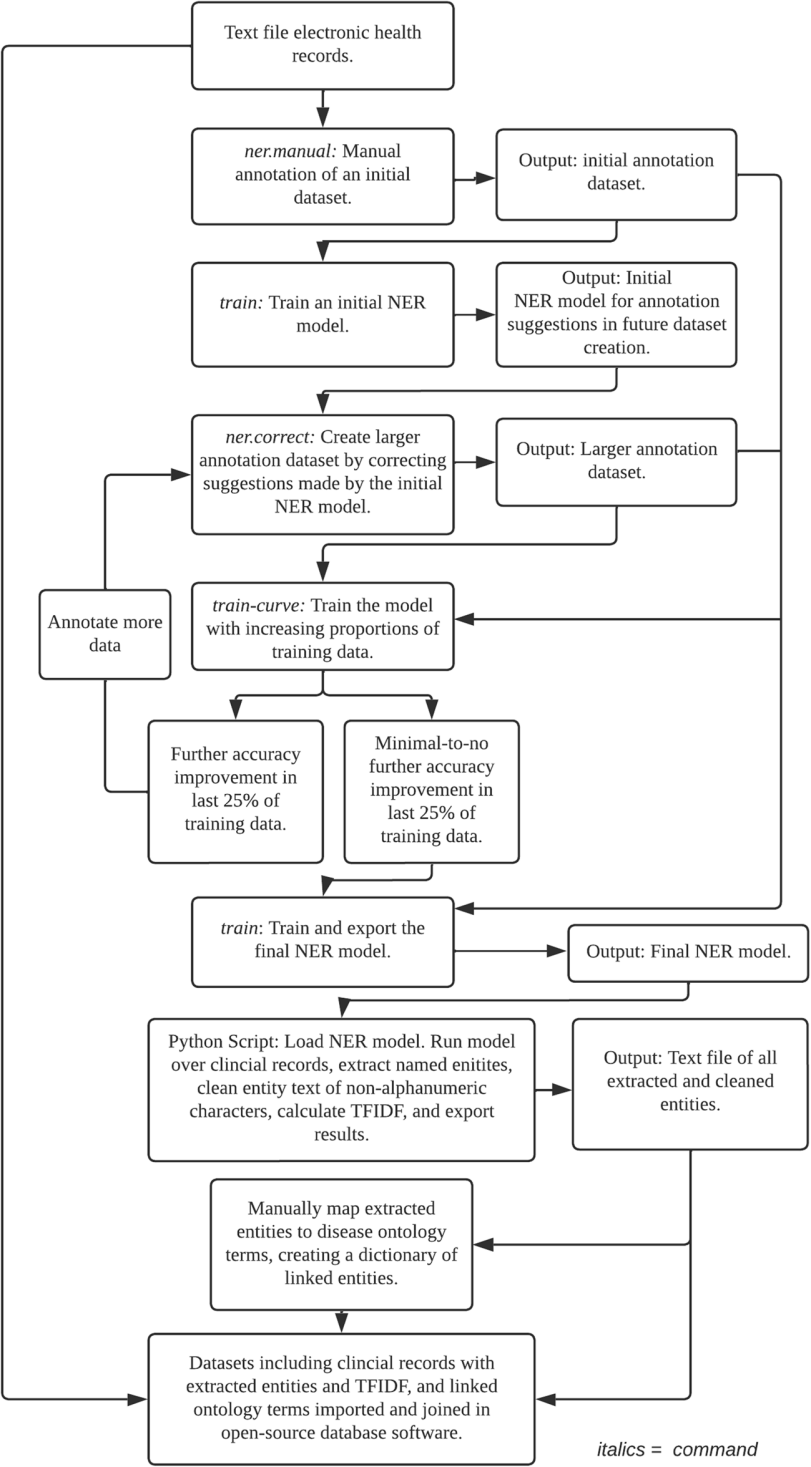
Summary of the workflow to build the low-code automated ophthalmic disease registry

We performed dataset annotation and NER model training using a low-code annotation software tool (*Prodigy*, ExplosionAI GmbH, Berlin, Germany) [20]. Prodigy is an active learning-based annotation tool and integrates with the spaCy natural language processing learning library. The architecture of the spaCy model is not open source but is described as using sub-word features, Bloom embeddings, and a deep convolutional neural network with residual connections. The tool enables the annotation of diagnoses by highlighting text in a graphical user interface displayed in a web browser (Fig. 1) [21]. The tool uses simple, one-line text commands entered into the terminal to execute tasks. These tasks are pre-scripted Python functions that initialise dataset annotation and train NER models. Figure 2 summarises the workflow.

Annotation was performed by a single qualified medical practitioner investigator with graduate ophthalmic experience (CM). Only ophthalmic diagnostic entities were annotated (Fig. 1). Non-ophthalmic diagnoses listed in past medical history when this occurred were not annotated. The spans of words containing the complete description of the diagnosis were annotated to ensure extractions were interpretable, non-ambiguous, and preserved a contextual window on either side of the diagnosis.

The annotation command tokenises the electronic clinical records into words to prevent errors of partial selection when annotating. Using the graphical user interface, we annotated the first 1000 health records to create an initial dataset of annotations (Fig. 2). We annotated only words relevant to the diagnosis, annotating multiple-word diagnoses as a complete annotation. Using the initial annotation dataset, we trained an initial NER model, which we subsequently used to provide suggested annotations in further dataset annotation to increase annotation efficiency.

A further and larger annotation dataset was created by annotating a proportion of the remaining clinical records and correcting the suggestions made by the initial NER model. We included only new records not previously annotated to create this dataset. We calculated accuracy statistics at approximately 500 note intervals by training a model using increasing proportions (25%, 50%, 75%, 100%) of the total annotations. Annotation of the clinical records continued until model accuracy showed minimal-to-no further improvement within the last 25%, occurring at 1923 records.

Using the low-code tool, we trained a final NER model using both the initial and larger annotation datasets. The model evaluation metrics included precision, recall, and standard *F* score [22]. The model training command reserves a proportion of annotations to evaluate the model and produce accuracy statistics after training. Therefore, creating a separate gold standard evaluation dataset is not required to evaluate the model’s performance. We used 20% of the annotations to produce the precision, recall, and *F* score. Precision refers to the ratio of true positives to the sum of true and false positives (TP/TP + FP), and recall refers to the ratio of true positives to the sum of true positives and false negatives (TP/TP + FN). NER model errors were analysed by the proportion of complete false positives, complete false negatives, and right label with overlapping span, as presented by Nejadgholi et al. [23].

To extract the diagnostic entities, we used the *spaCy* (v3.1.4) library to load and run the model over the entire set of clinical records. After extraction, regular expressions cleaned the entities to remove capitalisation and non-alphanumeric characters. In addition, we used the *gensim* (v4.1.2) library to calculate the term frequency-inverse document frequency (TF-IDF) for each entity-document pair to include for use in the registry. A binary weight was used for the term frequency and pivoted unique normalisation for document length normalisation. We used a binary weight as only the appearance of the entity in the document was relevant. Pivoted unique normalisation was used to counter bias introduced by document length and align the probabilities of retrieval and relevance [24], given that clinical notes can vary in length.

**Fig. 3:**
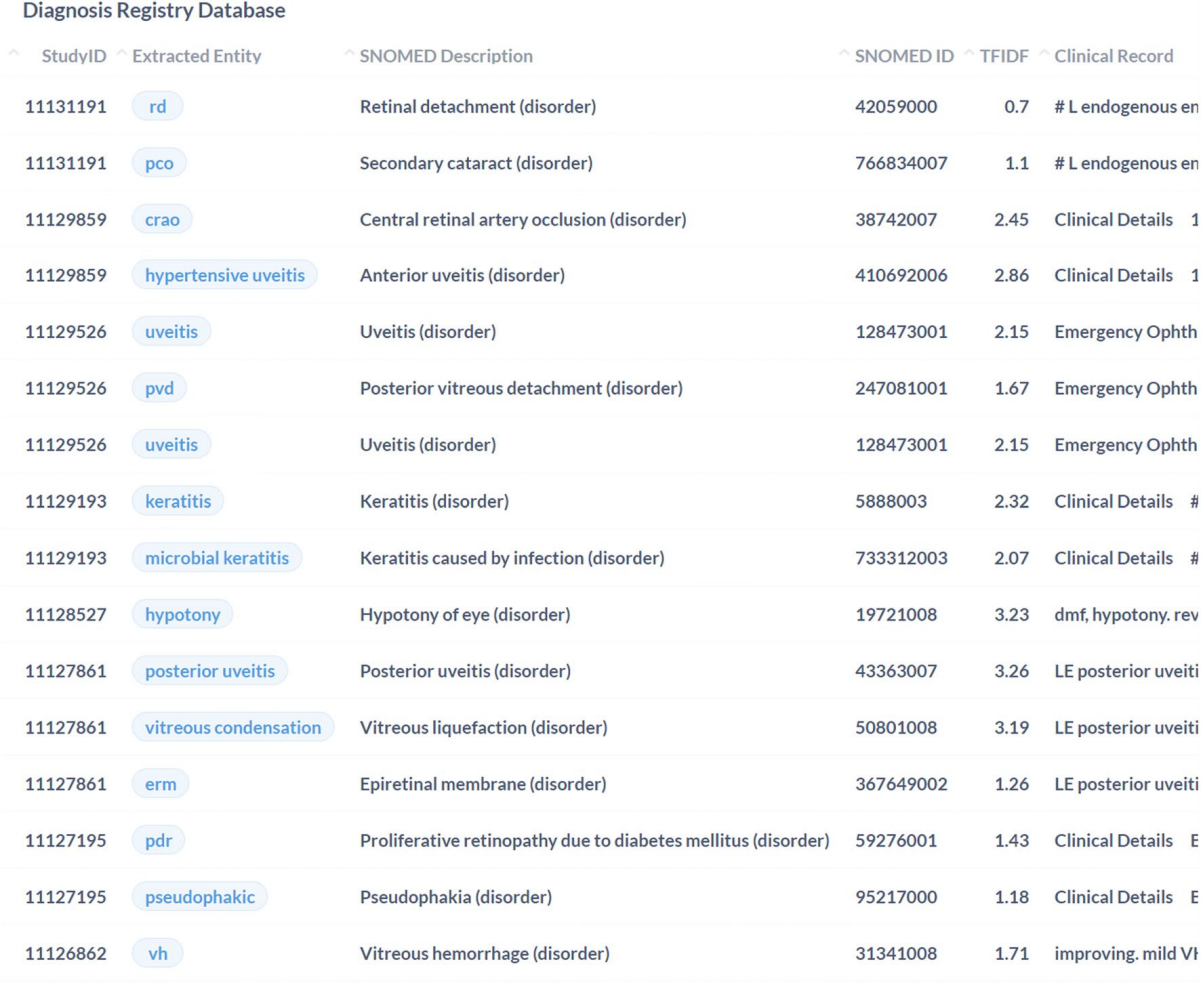
Interface of the automated ophthalmic disease registry

 We manually mapped a proportion of extracted entities representing common terms to SNOMED-CT (International Edition, version 2021-07-31) terms and corresponding codes. The datasets, including the clinical records, extracted entities, and their mapped SNOMED-CT terms, were imported into a free and open-source database management tool (*Metabase*, San Francisco, CA, USA) [25]. Datasets were joined via common data elements to produce a final registry containing patient medical record numbers, health records, extracted entities, and linked SNOMED-CT terms (Fig. 3).

We have condensed the steps for creating this registry into a series of sequential batch files (text files that execute a sequence of commands) for simple reproduction in any institution. Users must supply their electronic records to build the registry using our pre-trained NER model. Alternatively, users can train an institution-specific NER model in place of this using a variety of the available low-code annotation tools [26]. The reproducible registry files are hosted on GitHub (https://github.com/OphRL/AutoRegistry) along with instructions.

## Results

The model achieved an *F* score of 0.8128, precision (ratio of true positives to the sum of true positives and false positives) of 0.8157, and recall (ratio of true positives to the sum of true positives and false negatives) of 0.8099. The model was run over 33,455 notes, and a total of 123,194 named entities were extracted, 5070 of which were distinct (after decapitalisation and removing non-alphanumeric characters). The most frequently extracted diagnostic entities included ‘cataract’ (5.2%), followed by ‘ppv’ (3.0%), ‘erm’ (2.8%), ‘rd’ (2.3%), and ‘pseudophakic’ (2.2%). The 20 most frequent extractions are presented in Table 1.

**Table 1:** Most frequent entities extracted from text (decapitalised and non-alphanumeric characters removed)

Extracted entity	Number	Proportion of total entities (%)
cataract	6419	5.2
ppv	3744	3.0
erm	3476	2.8
rd	2887	2.3
pseudophakic	2727	2.2
cataracts	2533	2.1
iol	2296	1.9
phaco	2240	1.8
cmo	1956	1.6
poag	1940	1.6
pdr	1918	1.6
vh	1893	1.5
glaucoma	1746	1.4
pvd	1592	1.3
trab	1385	1.1
avastin	1382	1.1
pterygium	1367	1.1
dmo	1284	1.0
cnvm	1256	1.0
prp	1204	1.0

**Fig. 4:**
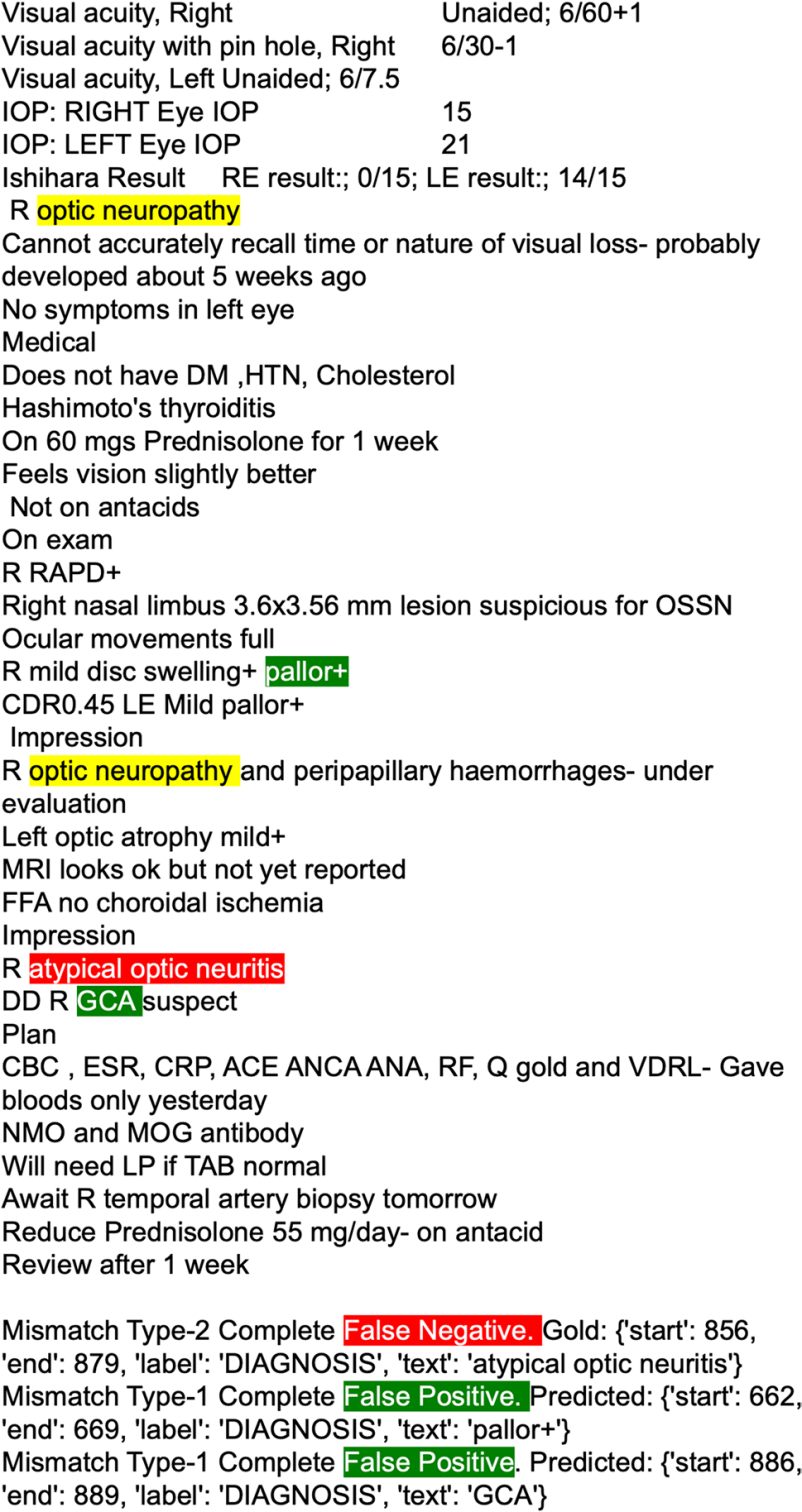
Example of clinical record with the NER model applied showing correct predictions, false negatives, and false positives. Yellow: correctly predicted diagnostic entities. Red: false negatives. Green: false positives

There were 159 type one (complete false positives), 102 type two (complete false negatives), and 20 type five (right label, overlapping span) mismatches. Figure 4 illustrates an example of a note containing correctly predicted diagnostic entities (yellow), false negatives (red), and false positives (green). The figure shows the correct labelling of ‘optic neuropathy’. However, the model did not predict the diagnostic entity ‘atypical optic neuritis’, resulting in a false negative. In addition, the model predicted the listed differential ‘GCA’ as a diagnostic entity which was recorded as a false positive.

Table 2 shows examples of lexical representations of cranial nerve palsies in the clinical records. The entities exemplify misspellings, abbreviations, acronyms, varying forms for the same concept, variable representation of numbers using words, and Arabic and Roman numerals.

**Table 2:** Examples of the various lexical representations of cranial nerve palsies in ophthalmic clinical records (decapitalised and non-alphanumeric characters removed)

Concept	Entities
Cranial nerve palsy	cn palsy, craneal nerve palsy, cranial nerve palsy
3rd cranial nerve palsy	3rd cn palsy, 3rd nerve palsy, cn iii microvascular palsy, cn iii palsy, cn3 palsy, cn3fourth palsy, cniii palsy, iii cn palsy, iii n palsy, iii nerve palsy, microvascular third nerve palsy, third nerve palsy, third nerve palsy suspect, total cn3 palsy
4th cranial nerve palsy	cn 4 palsy, cn 4th palsy, cn iv palsy, cn3fourth palsy, cn4 palsy, cniv palsy, congenital cn4 palsy, forth nerve palsy, fourht nerve palsy, fourth n palsy, fourth nerve palsy, fourth nerve paresis, iv cn palsy, iv n palsy, iv nerve palsy, iv palsy
5th cranial nerve palsy	cn v palsy, cn5 palsy, trigeminal nerve palsy
6th cranial nerve palsy	6th nerve palsy, 6th palsy, abducens nerve palsy, abducens palsy, acute cn vi palsy, cn 6 palsy, cn 6th palsy, cn vi palsy, cn6 new palsy, cn6 palsy, cnvi palsy, cranial nerve vi palsy, traumatic cn vi palsy, vi and vii palsy, vi cn, vi cn palsy, vi cranial nerve palsy, vi n palsy, vi n paresis, vi nerve palsy, vi nerve paresis, vi palsy, vith cnp, vith cranial nerve palsy, vith nerve palsy
7th cranial nerve palsy	bell’s palsy, bells palsy, branch viin palsy, cn 7 palsy, cn vii, cn vii palsy, cnvii palsy, facial n palsy, facial nerve deficit, facial nerve palsies, facial nerve palsy, facial nerve paralysis, facial nerve static palsy, facial nerve weakness, facial palsy, facial vii palsy, parotid gland resection cn 7th palsy, total facial nerve palsy, vi and vii palsy, vii palsy, viith palsy

## Discussion

Using a low-code workflow, we trained a NER model with moderate precision (0.8157) and recall (0.8099) and overall performance (*F* score 0.8128) in extracting diagnoses from free-text clinical records. Most errors were due to false positives, followed closely by false negatives. Overlapping spans accounted for a small proportion (7.1%) of errors during evaluation. A higher false positive rate is unlikely to impact the functioning of an automated registry, given that the aim is to detect all possible diagnoses present. However, false negatives are an area of potential improvement. The false positive pictured in Fig. 4 shows an example of a prediction that was incorrect due to its context rather than an incorrect diagnostic entity. Given that differential lists are a common occurrence, this may contribute to the higher false positive rate.

The complexities of clinical natural language are demonstrated through examples of variable representations of cranial nerve palsies in free text (Table 2). These entity examples illustrate the presence of misspellings, abbreviations, acronyms, variable forms of similar concepts, and variable representations of numerical expressions in ophthalmic notes. Low-code NLP tools enable the rapid creation of a disease registry containing a broad range of diagnoses in free-text electronic clinical records without requiring extensive clinician input. We implemented this pipeline in a ready-to-use tool to implement this workflow in any institution to create a disease registry.

Low-code NLP tools aim to reduce the barriers to implementing new and advanced artificial intelligence-based techniques for entity recognition in clinical and research workflows. We performed annotation using a user-friendly graphical interface, which was initialised using simple commands in the terminal (the text-based interface which enables interaction with the computer’s files and directories). Given that annotated datasets are required for supervised learning techniques, an increasing number of annotation tools are now available to create these datasets efficiently [26]. Features such as annotation suggestions are important, given that pre-annotation has previously been shown to improve annotation speed [27].

Rule-based approaches to extracting entities may perform well in task and domain-specific applications but are time-consuming and task-specific and require significant domain expert input when compared. Previous applications of such techniques to disease registries have included the use of regular expressions (text pattern matching) [28], modified tools based on regular expressions [29], and NLP tools using pre-trained models augmented with rule-based techniques [30, 31] [32]. Matching entities through regular expressions requires intimate knowledge of the representation of entities in clinical text and pre-specification of the patterns to detect. This specification is time-consuming and inflexible. For example, designing regular expressions to detect all possible representations of cranial nerve palsies, as depicted in Table 2, is complex. There have been significant advancements in artificial intelligence-based techniques for clinical NER, particularly with the introduction of transfer learning and transformer-based models [15]. For example, Moquarrab et al. presented a novel deep learning-based technique to extract clinical entities from clinical notes in the i2b2 NLP challenge datasets [33]. The authors used a combination of techniques, including a convolutional neural network, bidirectional long short-term memory (Bi-LSTM), and conditional random fields with non-complex embeddings. They achieved an *F*1 score of 93.57 and 86.11 across the 2010 and 2012 i2b2 datasets, respectively, showing significant improvements above previous applications. For comparison, the combination of the Bi-LSTM model and bidirectional encoder representations from transformers (BERT) embeddings achieved an *F*1 score of 90.25 and 80.91 in the i2b2 2010 and 2012 datasets, respectively [34]. Other popular models for NER, such as the conditional random field, achieved an *F*1 score of 84.30 in the i2bs 2010 dataset [35]. While it is difficult to perform comparisons across studies due to differences in pre-processing, dataset, and methodological differences, the benefits and improving performance of artificial intelligence-based techniques for clinical NER are promising for applications in automated registry production. However, few tools ready for implementation are currently available [15].

An ophthalmic disease registry could play an important role in identifying and monitoring rare diseases through electronic health records. It is estimated that 263–446 million persons are affected by rare diseases globally at any time [36]. Despite the clear burden of rare diseases and the need for research, rare disease research is limited by recruitment and sample size issues [37]. Searching diagnostic codes for instances of rare diseases is restricted by underrepresentation in most common ontologies such as the International Classification of Diseases [38] [39]. Electronic health records have been used previously to identify rare diseases [40, 41]; however, approaches to detection relied on regular expressions [42, 43]. A NER registry approach eliminates the pre-specification of expressions and is not diagnosis-specific, allowing flexibility in the range of diseases to be monitored. DeLozier et al. previously developed a system to monitor rare diseases through electronic health records [43]. An email alert system was used to prompt investigators to review rare drug reactions in clinical notes to improve recruitment in prospective clinical trials of drug-induced torsades de pointes and Stevens-Johnson Syndrome and toxic epidermal necrolysis. The alert system increased the rate of recruitment and reduced the time to enrolment in the studies. Monitoring diseases in free-text fields via integration with alerting systems can improve the monitoring of rare diseases and reduce barriers to cohort identification for research.

Diagnoses in unstructured free-text fields of electronic health records supplement manually coded diagnoses. The median accuracy of diagnostic coding in discharge summaries is 80.3% [44], but the coding of comorbidities in problem lists is often incomplete [45–48]. The lack of completeness results in poor sensitivity of diagnostic coding, despite achieving high specificity [45, 49–52]. Therefore, the absence of a diagnostic code does not necessarily reflect the absence of the disease. Coding accuracy is further affected by changes in the coding systems used [47], lack of suitably granular codes [53], incomplete coding in single centres due to data fragmentation across multiple sites [54], and length of time registered in an EHR [55]. Supplementing diagnostic coding with unstructured fields can improve this sensitivity [2, 56, 57]. This increased sensitivity has important implications for the case-finding ability of studies using electronic health records.

Our workflow has several limitations. The NER model extracts entities as they appear in text and is not integrated with a linking process to standard ontology. Therefore, linking terms to an ontology is considered a downstream task. However, building a database of diagnostic entities as they appear in the clinical records can inform further development of linking strategies or vocabulary databases. Our model was trained and evaluated using clinical records from a single institution. The model’s performance, if evaluated using external notes, is likely to be lower. However, rapid dataset annotation using low-code NLP tools means any institution can create custom NER models. Furthermore, annotations were performed by a single annotator. Thus, the registry represents the annotating characteristics of a single annotator. Multiple annotators may reduce this bias; however, annotators should be trained to follow annotation guidelines to ensure adequate inter-annotator agreement [57]. Lastly, all annotations were performed in English. Replication of the study findings with non-English free text would be beneficial.

We demonstrated a workflow using low-code NLP tools to produce an ophthalmic disease registry, with an accompanying ready-to-use tool to reproduce the registry in any institution. Our NER model displayed a moderate overall ability to extract ophthalmic diagnoses from free-text electronic clinical records. There is a further need for standard ophthalmic datasets for the evaluation of NER models and ready-to-use tools to encourage increased use of artificial intelligence for clinical NER tasks.
